# Direct Metal Laser Sintering Titanium Dental Implants: A Review of the Current Literature

**DOI:** 10.1155/2014/461534

**Published:** 2014-12-01

**Authors:** F. Mangano, L. Chambrone, R. van Noort, C. Miller, P. Hatton, C. Mangano

**Affiliations:** ^1^Department of Surgical and Morphological Science, Dental School, University of Varese, Via Giuseppe Piatti 10, 21100 Varese, Italy; ^2^ITEB Research Center, Dental School, University of Varese, Via Giuseppe Piatti 10, 21100 Varese, Italy; ^3^Dental Research Division, Department of Periodontology, Guarulhos University, Praca Teresa Cristina 229, 07023070 Guarulhos, SP, Brazil; ^4^Academic Unit of Restorative Dentistry, School of Clinical Dentistry, University of Sheffield, 19 Claremont Crescent, Sheffield S10 2TA, UK; ^5^Academic Unit of Oral and Maxillofacial Medicine and Surgery, School of Clinical Dentistry, University of Sheffield, 19 Claremont Crescent, Sheffield S10 2TA, UK

## Abstract

*Statement of Problem*. Direct metal laser sintering (DMLS) is a technology that allows fabrication of complex-shaped objects from powder-based materials, according to a three-dimensional (3D) computer model. With DMLS, it is possible to fabricate titanium dental implants with an inherently porous surface, a key property required of implantation devices. *Objective*. The aim of this review was to evaluate the evidence for the reliability of DMLS titanium dental implants and their clinical and histologic/histomorphometric outcomes, as well as their mechanical properties. *Materials and Methods*. Electronic database searches were performed. Inclusion criteria were clinical and radiographic studies, histologic/histomorphometric studies in humans and animals, mechanical evaluations, and *in vitro* cell culture studies on DMLS titanium implants. Meta-analysis could be performed only for randomized controlled trials (RCTs); to evaluate the methodological quality of observational human studies, the Newcastle-Ottawa scale (NOS) was used. *Results*. Twenty-seven studies were included in this review. No RCTs were found, and meta-analysis could not be performed. The outcomes of observational human studies were assessed using the NOS: these studies showed medium methodological quality. *Conclusions*. Several studies have demonstrated the potential for the use of DMLS titanium implants. However, further studies that demonstrate the benefits of DMLS implants over conventional implants are needed.

## 1. Introduction

Osseointegration, defined as a direct structural and functional connection between ordered, living bone and the surface of a load-carrying implant, is critical for implant stability and is considered a prerequisite for implant loading and long-term clinical success of endosseous dental implants [1, 2]. Since implant surface properties have long been identified as an important factor to promote osseointegration [1, 2], research has focused on optimizing the potential for osseointegration, and surface modifications have been extensively investigated [3–9].

Nowadays, dental implants are fabricated by machining titanium rods, followed by modification of the implant surface design, such as sandblasting [3], acid-etching [4, 5], anodization [6, 7], discrete calcium-phosphate crystal deposition [8], and chemical modification [3, 6, 9]. These have all been used to improve stability and enhance osseointegration [1–9]. In fact, several studies have demonstrated that implant surface topography plays a pivotal role in many peri-implant cellular and molecular mechanisms [3–10]. Rough surfaces have demonstrated better adsorption of biomolecules from biological fluids, which has the potential to alter the cascade of events that leads to bone healing and intimate apposition with the device [1–3, 10].* In vitro* reports indicate that rough surfaces improve the initial cellular response, including cytoskeletal organization and cellular differentiation with matrix deposition [1–3, 8, 9]. Histologically, it has been demonstrated that rough surfaces can effectively promote better and faster osseointegration, when compared to machined surfaces [11, 12]. From a clinical point of view, several studies have reported excellent long-term survival/success rates for rough surface implants [5, 7, 13].

However, all the aforementioned methods used for processing dental implants result in a high-density titanium structure with a micro- or nanorough surface. An alternative approach is to obtain implants with a functionally graded structure, possessing a gradient of porosity perpendicular to the long axis and relatively high porosity at the surface [14, 15].

In the last years, macroporous structures have become popular strategies to reach a tough and durable bone fixation [16]. In accordance with the modern concepts of bone tissue engineering, an open interconnected porous structure with pores in the range of 200–400 *μ*m is required for bone regeneration; in fact, extensive body fluid transport through the porous scaffold matrix is possible, which can trigger bone ingrowth, if substantial pore interconnectivity is established [16]. Improved fixation can be achieved by bone tissue growing into and through a porous matrix of metal, bonding in this way the implant to the bone host; however, from the mechanical point of view, this porous structure should be stiff enough to sustain mechanical loads [16, 17].

Since osseointegration is such an important factor in the success of dental implants, it may be biologically beneficial to use porous implants, extending the features that promote osseointegration beyond the surface, throughout the body of the device [18]. A variety of methods have been developed in recent years to produce a porous coating on the implants [19], with the specific aim of creating a structure capable of enhancing osseointegration, such as plasma spraying [20], three-dimensional fiber deposition [21], powder metallurgy [22], solid-state foaming techniques [23], and polymeric sponge replication [24].

With these conventional methods, however, it is impossible to fabricate a porous structure with a completely controlled design of the external shape as well as the internal pore network, with the tight constraints of porosity, optimum pore size, and mechanical strength that are required [25, 26].

As the development of open porous structures has been hampered by limitations in production techniques [19–24], there is a demand for fabrication methods for bulk porous titanium that can control porosity, pore size and distribution, and mechanical properties [14, 15, 25, 26].

Additive manufacturing (AM) methods have been proposed as a way to overcome this issue [14, 15, 25–27]. Additive manufacturing (AM), also known as solid freeform fabrication (SFF), layered manufacturing (LM), or direct digital manufacturing (DDM), is a strategy to generate directly physical objects with defined structure and shape on the basis of virtual three-dimensional (3D) model data [25–27]. In particular, AM comprises a group of techniques that can generate a physical model directly from computer-assisted-design (CAD) data or data provided by computer-based medical imaging technologies in a layer-by-layer manner, where each layer is the shape of the cross section of the model at a specific level [25–28].

Direct metal laser sintering (DMLS) is a laser-based AM technique, in which an object is built layer by layer using powdered metals, radiant heaters, and a computer-controlled laser [25–29]. Basically, the machine produces the object on a moveable platform by applying incremental layers of the pattern material [25–29]. For each layer, the machine lays down a film of powders, with an accurate thickness (0.1 mm). Then, a high power laser beam is directed on a powder bed and programmed to fuse metal powders present in its focal zone, according to a computer-assisted-design (CAD) file, thus generating a thin metal layer. The platform moves down the preprogrammed layer thickness, a fresh film of powder is laid down, and the next layer is melted with exposure to the laser source, so that it conforms to the previous layer. This process continues, layer by layer, until the object is fabricated [25–29].

With DMLS it is possible to control the porosity of each layer but also pore interconnectivity, size, shape, and distribution, and consequently the 3D architecture of the implant, by changing the processing parameters, such as laser power and peak power (for CW and pulsed lasers, resp.), laser spot diameter, layer thickness, hatching pitch (or scan spacing), scan speed, and scanning strategy, or by modifying the size of the original titanium particles [25–29]. This is an important advantage of this technique: a high level of interconnectivity resulting in a predominantly open pored morphology may allow bone ingrowth and vascularization, thus enhancing osseointegration, the essential factor of the long-term reliability of an implant [25–29]. In addition, since the mechanical properties of biomaterials are dependent on their microarchitecture, DMLS technology can be used to fabricate porous titanium implants with mechanical properties (stiffness) close to bone [25–29]. In fact, a porous implant will have a yield strength and elastic modulus that are reduced compared to a fully dense component. As a result, the mechanical properties of a porous device can be tailored to better match the yield strength and elastic modulus of the host bone and therefore avoid undesired effects such as “stress shielding” which is associated with a mismatch in bone-implant elastic moduli [25–29]. Another advantage of DMLS is to have the unlimited freedom and ability to fabricate complex-shaped patient-specific, custom-made titanium implants, in a cost-time competitive manner [28, 29]. In fact, as DMLS builds up parts directly from CAD data, no further tooling costs or inventories are necessary. Finally, in contrast to cutting or milling processes, DMLS technology produces less waste and thus there is almost no loss of material [28, 29].

Even though by now the concept of AM and DMLS technologies for implant and biomaterial manufacturing are well accepted, there is still limited data available on DMLS titanium implants in the current literature. The aim of the present review was to evaluate the evidence for the reliability of DMLS titanium dental implants and the clinical and histologic/histomorphometric outcomes, as well as their mechanical properties.

## 2. Materials and Methods

### 2.1. Study Design

The protocol of this review is in accordance with PRISMA (Preferred Reporting Items for Systematic Reviews and Meta-Analyses) [30], the Cochrane Collaboration [31], and CheckReview [32] checklists. It was developed a priori and covered all aspects of review methodology: rationale, design, focused question, inclusion and exclusion criteria, search strategy, quality assessment, and data synthesis. The focused questions read: “What is the reliability of DMLS titanium dental implants? May the use of DMLS implants provide benefits, in terms of clinical outcome and histologic/histomorphometric results, compared to the use of conventional implants?”.

### 2.2. Study Samples and Variables

The protocol recognized that randomized controlled trials (RCTs) are the most appropriate designs to address a focused question that embraces effectiveness of interventions. However, both experimental (RCTs) and observational studies (case reports, case series, and case-control and prospective cohort trials) were included in the hierarchy of evidence for this review. Inclusion criteria were clinical and radiographic studies, histologic and histomorphometric studies in humans and animals, mechanical evaluations, and* in vitro* studies (cell cultures) on DMLS titanium implants. Exclusion criteria were studies in which there was no reference to either the treating or supervising clinician or the site (practice/hospital/university) where the research was done.

### 2.3. Search Strategy

Electronic database searches of MEDLINE, EMBASE, and SCOPUS were performed using keywords and MeSH terms based on a search strategy used for searching MEDLINE (via PubMed): (((direct metal laser sintering implants OR direct metal laser forming implants OR selective laser sintering implants OR selective laser melting implants)) OR (additive manufacturing implants OR laser-sintered implants OR direct laser fabrication implants OR porous titanium implants OR porous titanium scaffolds)) AND (dental implant OR (dental AND implant) OR osseointegration). The searches were confined to full-text articles written in all languages since and including January 2005 to January 2014 presenting either clinical/radiographic data, histologic/histomorphometric evaluations, mechanical evaluations, or* in vitro* (cell cultures) studies. Titles and abstracts were screened and then full texts of all potentially relevant publications were obtained and reviewed independently in duplicate by F. Mangano and L. Chambrone, who also undertook data extraction. The purposely designed data extraction forms recorded study title, authors, type of study, randomization and blinding if present, number of subjects treated, study design and treatment phase, follow-up, outcomes, statistical findings, and conclusions. In addition, the reference lists of included studies were also hand-searched.

### 2.4. Assessment of Risk of Bias/Quality Assessment in Included Human Studies

For RCTs, the methodological quality of the studies was designed to assess the points described by the Cochrane Collaboration's tool for assessing risk of bias [31] and detailed in previous publications: (1) method of randomisation; (2) allocation concealment; (3) masking of examiners with regard to the treatment; (4) completeness of the follow-up; (5) selective reporting; and (6) other sources of bias.

For observational studies, the Newcastle-Ottawa scale (NOS) [33] adapted by Chambrone and colleagues [34] was used to evaluate the methodological quality of all studies included (Appendix). Concisely, the subsequent topics were evaluated: (a) selection of study groups (i.e., sample size calculation, representativeness of the patients and their selection, ascertainment/assessment of peri-implant conditions, clear description of methods used for DMLS, training/calibration of assessors of outcomes, data collection, and description of clear inclusion/exclusion criteria); (b) comparability (i.e., comparability of patients on the basis of the study design or analysis and management of potential confounders); (c) outcome (i.e., evaluation of results, assessment of peri-implant outcomes and adequacy of follow-up of the patients); and (d) statistical analysis (i.e., appropriateness/validity of statistical analysis and unit of analysis reported in the statistical model). Also, stars (points) were given to these methodological quality criteria, and each study included could receive a maximum of 14 points. Studies with 11–14 stars (approximately 80% or more of the domains satisfactorily fulfilled) were arbitrarily considered as being of high quality, with 8–10 stars indicating medium quality and <8 stars suggesting low methodological quality.

### 2.5. Statistical Analysis/Meta-Analysis

Meta-analysis was considered only if it was possible to find randomized controlled studies (RCTs) with an outcome measurement of histologic evaluation with histomorphometric analysis reported. Mean and standard deviation (SD) values of newly formed bone from each study would have been used, and weighted mean values would have been assessed to account for the difference in the number of subjects among the different studies. To compare the results between the test and the control groups, the differences of regenerated bone in mean and 95% confidence intervals (CIs) would have been calculated, with the aid of a statistical package (Package Metafor; Wolfgang Viechtbauer, Maastrich, The Netherlands).

## 3. Results

### 3.1. Results of Search and Included Studies

**Figure 1:**
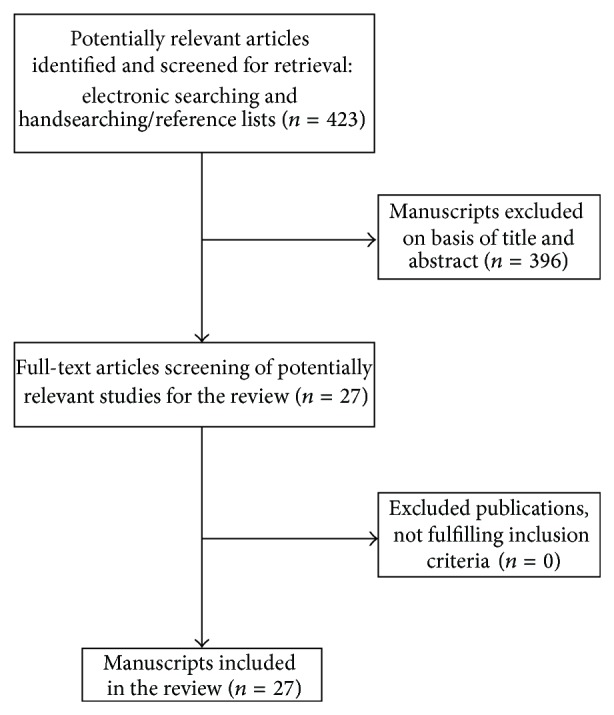
Flow chart of manuscripts screened through the review process.

Of the 423 potentially eligible publications initially identified by the search strategy, 396 were excluded following review of the title and/or abstract. In total, 27 studies were considered to be eligible for inclusion in the present literature review (Figure 1). These articles were published over a 10-year period, between 2005 and 2014, and demonstrated considerable variation with respect to study type, study design, follow-up, and results. No RCTs on DMLS implants were found in the current literature. Among all the works included in the present review, 7 were clinical studies (4 cohort studies; 1 case series; 2 case reports) reporting results of only test groups without any control groups; 6 were histologic/histomorphometric studies on humans (3 CTs; 3 case series); 6 were histologic/histomorphometric studies on animals (5 CTs; 1 case series); 4 were mechanical studies; and finally, 4 were cell culture studies.

### 3.2. Clinical Studies on DMLS Titanium Implants

Only a few clinical studies on DMLS titanium implants were found in the current literature [35–41]. Among these, 3 were prospective clinical studies on standard size dental implants (Tixos, Leader Implants, Milan, Italy) [35–37], while 4 were clinical studies or reports on the application of DMLS technology for the fabrication of custom-made implants [38–41]. All the implants used in these studies were fabricated with the same processing parameters/laser settings and were characterised by a porous surface with an average pore size of 200–400 *μ*m, on a bulk, dense titanium core [35–41]. In the first prospective multicenter clinical study using internal-hexagon DMLS implants, a total of 201 implants (106 maxillae, 95 mandibles) were inserted in 62 patients, with a two-stage technique (the healing time was 2-3 months in the lower jaw and 3-4 months in the upper jaw) [35]. The prosthetic restorations comprised 105 single crowns (SCs), 45 fixed partial prostheses (FPPs), and two fixed full-arch prostheses (FFAs). At the end of the study, after 1 year of functional loading, an overall implant survival rate of 99.5% was reported, with only one implant loss (maxilla: 99.0%, 1 implant failure; mandible: 100.0%, no implant failures) [35]. Among the survived implants (200), 5 did not fulfill the established clinical and radiographic success criteria, giving an implant-crown success of 97.5%. Finally, the mean distance between the implant shoulder and the first visible bone contact (DIB) was 0.4 mm (±0.2). This study supports the concept that internal-hexagon DMLS implants can be used in fixed prosthetic rehabilitations of both jaws, with a predictable positive outcome [35]. In another prospective study on the immediate loading of mandibular overdentures supported by unsplinted, one-piece ball attachment DMLS implants, with 96 implants inserted in the edentulous mandible of 24 patients, a satisfactory 1-year implant survival rate of 98.9% was reported [36]. Only one implant was lost. Among the surviving 95 implants, 2 did not fulfill the established clinical and radiographic success criteria, for an overall implant success rate of 97.8%. The mean DIB was 0.2 mm (±0.3; 95% confidence interval: 0.24–0.32) [36]. Based on these results, the authors concluded that the immediate loading of unsplinted DMLS implants by means of ball attachment-supported mandibular overdentures seems to represent a safe and successful procedure [36]. Finally, in a 2-year prospective clinical study on the immediate restoration of fixed partial prostheses (FPPs) supported by one-piece narrow-diameter (2.7–3.2 mm) DMLS implants, where 37 implants were installed in the posterior jaws (14 maxillae, 23 mandibles) of 16 patients, no implant failure occurred, resulting in a 100% survival rate [37]. The implant success, based on clinical and radiographic criteria, was 94.6%, and the mean DIB was 0.4 mm (±0.3) [37]. This study supports the hypothesis that one-piece narrow-diameter DMLS implants can be successfully used in fixed prosthetic rehabilitations in the posterior regions of both jaws [37]. Finally, with DMLS, patient-specific implants can be produced: in fact, this technique can be used for the fabrication of custom-made titanium implants, such as root-analogues [38–41] or blade implants [42], adapting the implant to the anatomy of the patient instead of adapting the patient's bone to a preformed standardized fixture. Two different case reports have demonstrated that modern cone beam computed tomography (CBCT) acquisition and 3D image conversion, combined with the DMLS process, allow the fabrication of custom-made, root-analogue implants [39, 40]. In these reports, CBCT images of residual nonrestorable roots of maxillary premolars (biradicular first premolar and monoradicular second premolar, resp.) were acquired and modified with specific software into 3D models. From these models, two custom-made, root-analogues DMLS implants were fabricated, as perfect copies of the radicular units that needed replacement. The nonrestorable residual roots were extracted, and immediately after extraction the root-analogues were placed into the extraction sockets and restored with single crowns [39, 40]. After 1 year of functional loading, the customized implants showed excellent integration in the bony tissue, with almost perfect functional and aesthetic outcome [39, 40]. After these first reports, a prospective clinical study evaluated the survival and success rate of DMLS, root-analogue implants, placed into the extraction sockets of 15 patients [41]. CBCT images of 15 nonrestorable premolars were acquired and transformed into 3D models: from these, custom-made, root-analogue DMLS implants with integral abutment were fabricated. Immediately after tooth extraction, the root-analogues were placed in the sockets and restored with single crowns. At the 1-year follow-up, no failures were reported. All implants were stable, with no signs of infection [41]. The optimal conditions of the peri-implant tissues were confirmed by the radiographic examination, with a mean DIB of 0.7 mm (±0.2) [41]. The authors concluded that the DMLS technique offers a novel and interesting perspective for the immediate placement of customized dental implants [41].

### 3.3. Histologic and Histomorphometric Evaluations on DMLS Titanium Implants

In total, 12 histologic/histomorphometric studies on DMLS implants were found in the current literature (6 were animal studies and 6 were human studies) [15, 17, 43–52].

#### 3.3.1. Animal Studies

The animal studies were conducted on DMLS titanium implants/scaffolds with different geometric characteristics and porosity [15, 17, 43–47]. van der Stok and colleagues [17] studied the biological response to two different titanium DMLS scaffolds with struts of 120 *µ*m (titanium-120) or 230 *µ*m (titanium-230) in a load-bearing critical femoral bone defect in rats. The defects were stabilized with an internal plate and treated with titanium-120 or titanium-230 or left empty [17].* In vivo* micro-CT scans at 4, 8, and 12 weeks showed more bone in the defects treated with scaffolds: 18.4 ± 7.1 mm^3^ (titanium-120, *P* = 0.015) and 18.7 ± 8.0 mm^3^ (titanium-230, *P* = 0.012) of bone were formed in those defects, significantly more than in the empty defects (5.8 ± 5.1 mm^3^) [17]. This study demonstrated that, in addition to adequate mechanical support, porous titanium scaffolds can facilitate bone formation, which results in high mechanical integrity of the treated large bone defects [17]. In another histologic/histomorphometric study, de Wild and colleagues [43] investigated the* in vivo* bone formation with different DMLS porous titanium implants (with their surfaces either left untreated, sandblasted, or sandblasted-acid etched) placed into calvarial bone defects in rabbits and compared to untreated defects. In this study, DMLS implants had an open porous lattice and a stepped cylindrical shape with an upper outer diameter of 7.5 mm, a lower outer diameter of 6 mm, and a height of 3.8 mm, while rod thickness was set at 200 *μ*m [43]. The open porous channels with a quadratic cross section of 700 *μ*m × 700 *μ*m were designed with an overall porosity of 83.5% [43]. At the end of the study, bone augmentation beyond the original bone margins was only seen in implant treated defects, indicating a high osteoconductive potential of the DMLS implants [43]. Analysis by *μ*CT and histomorphometry revealed that all the porous titanium structures were well osseointegrated into the surrounding bone [43]. However, the histomorphometric analysis revealed that bone formation significantly increased in the DMLS sandblasted implants compared to DMLS untreated ones and bone bridging was significantly increased in DMLS sandblasted-acid etched scaffolds, thus suggesting that scaffolds manufactured by DMLS should be surface-treated [43]. The authors concluded that designed porous, lightweight structures have potential for bone regeneration and augmentation purposes, particularly when patient-specific geometries are needed [43].

Fukuda and colleagues [44] tested the effects of interconnective pore size of titanium DMLS scaffolds on osteoinductivity and the bone formation processes. DMLS was employed to fabricate porous titanium scaffolds (diameter 3.3 mm, length 15 mm) with a channel structure comprising 4 longitudinal square channels, representing pores of different widths (500, 600, 900, and 1200 *μ*m, resp.). The DMLS scaffolds were implanted in the dorsal muscles of 8 mature Beagle dogs, remaining for periods of 16, 26, or 52 weeks [44]. Excellent osteoinduction was observed in scaffold with pores of 500–600 *μ*m [44]. This study supports the hypothesis that the geometric properties of the DMLS scaffolds (characterised by an open interconnective porosity with pores of controlled size) can give rise to new bone formation even in extraskeletal sites [44]. In a similar study, Pattanayak and colleagues [45] investigated the biological response to highly porous DMLS titanium scaffolds when installed into the femur of Japanese white rabbits. Twelve weeks after implantation, the histologic evaluation showed excellent osteoconductive properties for the DMLS scaffolds, with substantial amount of new bone penetrating into the pores and directly bonding to the walls within the implants [45]. Stübinger and colleagues [46] placed three different types of implants (machined, sandblasted-acid etched, and DMLS implants) in the pelvis of six sheep. In this case, the DMLS implants were characterised by a porous surface and a dense titanium core. After 2 and 8 weeks, bone-to-implant contact (BIC) values of the DMLS surface (2 weeks: 20.4% ± 5.1%; 8 weeks: 43.9% ± 9.6) revealed no statistical significant differences in comparison to the machined (2 weeks: 20.3% ± 11.5%; 8 weeks: 25.3% ± 4.6%) and sandblasted-acid etched (2 weeks: 43.6 ± 12.2%; 8 weeks: 53.3 ± 8.9%) surfaces [46]. However, removal-torque-tests showed a significant improvement in fixation strength (*P* < 0.001) for the DMLS (1891.8 ± 308.4 Nmm) surface after 8 weeks in comparison to the machined (198.9 ± 88.0 Nmm) and sandblasted-acid etched (730.0 ± 151.8 Nmm) surfaces [46]. Similar results were obtained in another study by Witek and colleagues [47], where DMLS and sandblasted-acid etched implants (one per type) were placed in the radius of 18 Beagle dogs, remaining for 1, 3, and 6 weeks (*n* = 6 dogs per evaluation time)* in vivo*. Again, the DMLS implants were porous in their surface only. BIC and removal torque were evaluated [47]. A significantly higher BIC was observed for DMLS implants (*P* < 0.04) only at 1 week, whereas no significant differences were found at 3 and 6 weeks; however, a significantly higher torque was observed at 1 (*P* < 0.02) and 6 weeks (*P* < 0.02) for the DMLS implants, whereas at 3 weeks no significant differences were observed [47]. The authors concluded that the DMLS implants presented biocompatible and osseoconductive properties and improved biomechanical response compared with the sandblasted-acid etched implants at 1 and 6 weeks* in vivo* [47].

#### 3.3.2. Human Studies

All human studies were based on DMLS titanium implants with a porous surface and a dense titanium core [15, 48–52]. Shibli and colleagues [15] investigated the influence of DMLS surface topography on bone-to-implant contact (BIC), on bone density in the threaded area (BA), and on bone density outside the threaded area (BD) in type IV bone after 8 weeks of unloaded healing. In total, 30 patients received 1 microimplant (2.5 mm diameter and 6 mm length) in the posterior maxilla. Thirty microimplants with three different topographies were evaluated: 10 machined; 10 sandblasted-acid etched surface (SAE), and 10 DMLS microimplants [15]. After 8 weeks, the microimplants and the surrounding tissues were removed and prepared for the histomorphometric examination. The histomorphometric analysis revealed that the mean BIC was higher for the DMLS and SAE surfaces (*P* = 0.0002) [15]. The BA was higher for the DMLS surface, although there was no significant difference with the SAE surface, while the BD was similar for all topographies (*P* > 0.05). The study suggested that the DMLS and SAE surfaces presented a higher BIC rate compared with machined surfaces under unloaded conditions, after a healing period of 8 weeks [15]. In another study by the same group of researchers [48], 4 DMLS microimplants were inserted in the posterior mandible of 4 patients. After 8 weeks, the microimplants and the surrounding tissue were removed and prepared for the histomorphometric analysis, scanning electron microscopy (SEM), and X-ray dispersive spectrometry (EDS) evaluation [48]. The histomorphometric evaluation revealed a mean BIC of 60.5 ± 11.6%. The SEM and EDS evaluation showed a close relation between newly formed bone matrix and DMLS surface, in accordance with the histological features [48]. This study confirmed that the DMLS surface can provide an optimal stratum to bone tissue ingrowth [48]. These results confirmed those of a previous report by Mangano and colleagues [49] where one DMLS microimplant was inserted in the anterior mandible of a patient, retrieved after 8 weeks of unloaded healing with the surrounding tissues, and prepared for histomorphometric analysis. Histologically, the peri-implant bone appeared in close contact with the implant surface, whereas marrow spaces could be detected in other areas along with prominently stained cement lines [49]. The mean BIC was 69.51% [49]. In another study by Shibli and colleagues [50], 12 totally edentulous patients received DMLS transitional implants in the posterior maxilla, 2 implants per patient. Twelve implants were immediately loaded, to support an interim complete maxillary denture during the healing period, while the other 12 were left unloaded. Eight weeks after surgery, the transitional implants and the surrounding tissue were removed and prepared for histomorphometric evaluation [50]. Histometric evaluation indicated that the mean BIC was 45.2 ± 7.6% and 34.1 ± 7.8% for immediate loaded and unloaded implants, respectively (*P* < 0.05). Immediately loaded DMLS implants in posterior maxilla showed higher BIC compared to unloaded implants [50]. Although these data must be considered with caution, because of the inherent limits of this study, both immediately loaded and unloaded DMLS implants showed a high BIC in the posterior maxilla [50]. Since X-ray micro-CT can provide rapid, nondestructive 3D images and measurements on bone microstructure, the interface between bone tissue and DMLS titanium implants has been studied accordingly [51]. In particular, high resolution micro-CT has been achieved with synchrotron radiation-based computed microtomography (SRmCT) [51]. Two DMLS titanium microimplants were inserted in the posterior maxilla of a patient and retrieved after 8 weeks. One of these implants was treated to obtain thin ground sections, for histological evaluation, whereas the other underwent a SRmCT evaluation [51]. The histological evaluation revealed a BIC of 65.2%: the newly formed bone was primarily composed of woven bone connecting the peri-implant bony trabeculae to the microimplant surface [51]. The implant surface showed superficial debris or particle inclusions in the surrounding tissue close to the bone area. These results were confirmed by SRmCT investigation [51]. Finally, a study evaluated the peri-implant soft tissues around human-retrieved DMLS microimplants [52]. Twenty-four microimplants were inserted in the posterior maxilla of 12 patients (two implants per patient). In order to evaluate the behaviour of the peri-implant soft tissues, the neck of the implants had two different surface topographies, DMLS topography (test group) and acid-etched surface topography only (control group) [52]. After 8 weeks, all the implants and the surrounding tissue were removed and prepared for histomorphometric evaluation. In the control specimens, collagen fibers were oriented perpendicular to the surface for a distance of 100 *μ*m, whereafter they became parallel, running in several directions. In the test specimens, a more intimate contact of the fibrous matrix with the implant surface was evidenced, with the collagen bundles more perpendicularly oriented to the DMLS surface [52]. Some collagen bundles were directly bonded to the DMLS surface. The authors concluded that, by changing the surface microtexture, it is possible to change the response of the peri-implant soft tissues [52].

### 3.4. Assessment of Risk of Bias/Quality Assessment in Included Human Observational Studies

**Figure 2:**
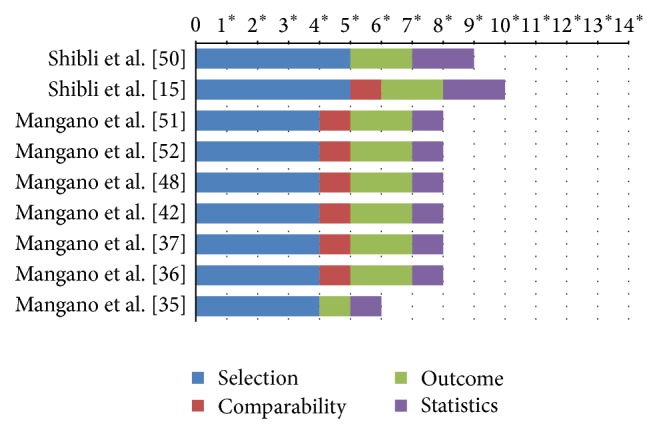
Methodological quality of included observational studies (stars assigned to respective study).

As reported previously, no RCT could be identified, and thus only the outcomes of prospective observational studies (e.g., case series, case-control, and prospective cohort trials) could be assessed. The methodological quality of included observational studies [15, 35, 37, 42, 48, 50–52] is depicted in Figure 2. All of these studies were considered to have a medium methodological quality, except for the paper by Mangano et al. [35] that was set as being of low quality.

### 3.5. Mechanical Studies on DMLS Titanium Implants

Titanium and its alloys are widely used for various implants, in the orthopaedic and dental fields, because of good corrosion resistance, high osteoconductivity, and mechanical strength [14, 27, 29, 44, 53–56]. However, Young's modulus of *α* (105 GPa for pure Ti) and *α* + *β* titanium alloys (110 GPa for Ti-6Al-4V) is about 3–10 times higher than that of bone (10–30 GPa). This mismatch of modulus between metallic implant and surrounding bone can cause “stress shielding” effects, which eventually lead to bone resorption [14, 27, 29, 44, 53–56]. In orthopaedics, bone resorption caused by stress shielding represents a major problem, as it is believed to contribute to highly undesired effects such as aseptic loosening of implants [27, 29, 44]. One approach for reducing stress shielding is to use porous metallic biomaterials: if a considerable amount of interconnected pores is introduced into them, their elastic moduli may significantly decrease [14]. In the study of Traini and colleagues, surface appearance, microstructure, composition, mechanical properties, and fractography of DMLS titanium implants were evaluated [14]. The results of the mechanical tests indicated that DMLS resulted in a “functionally graded” material, with a compact sintered titanium core (104 ± 7.7 Gpa) with a modulus similar to that of machined titanium, while the modulus of the porous titanium present on the implant surface was reduced (77 ± 3.5 Gpa) and more “similar” to that of bone. The authors concluded that DMLS implants may show better adaptation to the elastic properties of the bone [14]. Such implants could minimize stress shielding effects and improve long-term performance [14]. Sallica-Leva and colleagues [53] investigated the influence of the microstructure on the mechanical properties of DMLS implants. The authors concluded that the mechanical properties of the parts obtained by DMLS fall in a range that is interesting for bone substitution applications [53]. In addition, a comparison between these results and those of porous parts with similar geometry obtained by electron beam melting (EBM) technology showed that the use of DMLS allows parts with higher mechanical properties for a given relative density to be obtained [53]. Amin Yavari and colleagues [54] studied the fatigue behaviour of porous structures made of Ti6Al4V using DMLS. Four different porous microarchitectures were manufactured with high porosities (between 68% and 84%) and the fatigue S-N curves of these structures were determined. At the end of the study, the absolute S-N curves of these four porous structures were very different. In general, given the same absolute stress level, the fatigue life was much shorter for more porous structures [54]. The authors concluded that the normalized endurance limits of the tested structures were lower than that of solid titanium (with similar alloy) and that of some other porous titanium structures manufactured using other techniques [54]. Almeida and colleagues [55] investigated the mechanical behaviour of DMLS titanium dental implants. Step-stress accelerated life testing (SSALT) and fractographic analysis were performed to compare the reliability and failure modes of DMLS and sandblasted-acid etched (SAE) implants used for anterior single-unit replacements [55]. Forty-two standard dental implants (3.75 mm × 10.0 mm) were used; among these implants, 21 were fabricated with DMLS technology while the other 21 were SAE implants. The abutments were screwed to the implants and standardized maxillary central incisor metallic crowns were cemented and subjected to SSALT in water. At the end of the study, no differences in reliability and fracture mode were observed between DMLS and SAE implants used for anterior single-unit crowns [55]. These findings suggested that DMLS technology titanium implants may not affect the implant fatigue endurance [55].

### 3.6. Cell Cultures and Surface Characterization

**Figure 3:**
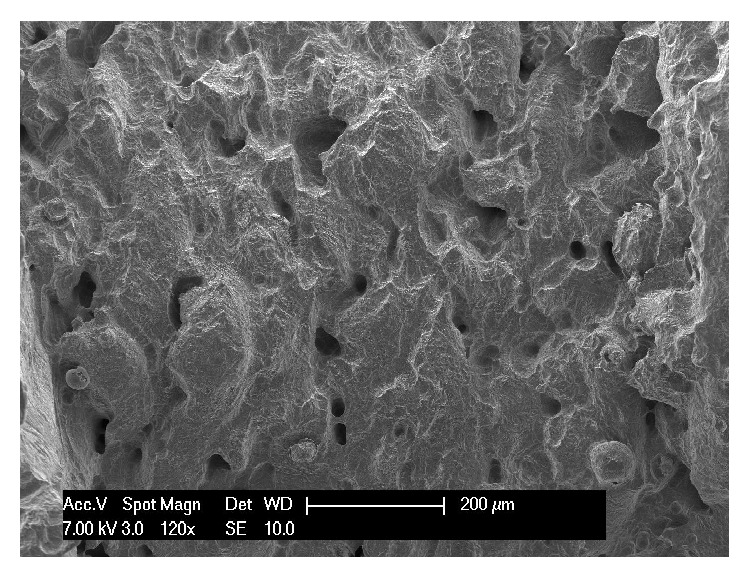
Scanning electron microscopy of the DMLS implant surface. The DMLS surface is irregular with ridge-like and globular protrusions, interspersed by intercommunicating pores and irregular crevices. The alternation of rounded features, narrow crevices, and deep indents is particularly evident (120x).

The surface produced with DMLS has been investigated and characterised using scanning electron microscopy (SEM) [14, 27, 28], stereo-scanning electron microscopy (stereo-SEM), [28] and atomic force microscopy (AFM) [56]. The SEM and stereo-SEM evaluations revealed a porous surface, with a pore network extending 200 *μ*m beneath the surface; the surface was characterised by deep, intercommunicating crevices, shallow depressions, and deep, rounded pits of widely variable shape and size (Figure 3) [14, 27, 28, 56]. The roughness parameters were Rt, 360.8 *μ*m; Rz, 358.4 *μ*m; Ra, 67.4 *μ*m; and Rq, 78.0 *μ*m [28]. This porous network with high values of microroughness may influence the shape that cells adapt within the 3D cavities, inducing a specific genetic expression [14, 16, 19, 27–29]. “The AFM evaluation evidenced that the DMLS surface geometry may represent a valid substratum for protein adsorption, consequently facilitating cell adhesion [56].” After the complete morphological characterization, studies on cell cultures have investigated the biological response to DMLS surface [27, 28, 57, 58]. In the first* in vitro* investigation, Hollander and colleagues [27] cultured human osteoblasts on porous blasted DMLS specimens to study morphology, vitality, proliferation, and differentiation of the cells, at 3, 7, and 14 days. At day 14, the cells were vital and proliferating. On porous specimens, osteoblasts grew along the rims of the pores and formed circle-shaped structures, as visualized by live/dead staining as well as SEM [27]. Some of the pores were completely filled with cells. This first* in vitro* experiment demonstrated that DMLS-fabricated Ti-6Al-4V allowed structure-oriented growth of human osteoblasts on its surface [27]. These results were confirmed by another study on cell cultures, where rat calvarial osteoblasts were seeded and cultured on disc specimens produced by DMLS [28]. After 9 days, cells had attached to and spread on the surface; they were irregularly shaped, predominantly attached to protruding features, and spanned across intervening crevices by means of extended tightly stretched processes. Higher magnification images showed that where a cell body or lamellipodium contacted the surface, it was closely adherent and occluded the microcavities beneath [28]. Cell density was similar to that on a commercial rough microtextured surface but lower than on machined and smooth-textured grit-blasted, acid etched surfaces [28]. In the same study, human fibrin clot extension on the DMLS surface was investigated. An extended fibrin clot covered the DMLS surface, creating a 3D network [28]. More recently, Matena and colleagues [57] analysed the proliferative behaviour of primary osteoblasts and an endothelial cell line when cultured on DMLS titanium scaffolds. The cells were stimulated with angiogenic factors (VEGF and HMGB1). The osteoblasts were able to proliferate and migrate on the DMLS titanium surface, and they could be visualized up to 210 *μ*m in depth of the pores. The authors concluded that the establishment of an* in vivo* model to evaluate the DMLS titanium scaffold appears to be promising [57]. Finally, in another* in vitro* study, human osteoblasts and stem cells derived from human dental pulps (dental pulp stem cells, DPSCs) were cultured either on acid-etched (AE) or on DMLS titanium surfaces, in order to investigate their osseointegration and clinical use capability of derived implants [58]. The cells were challenged with the two titanium surfaces, either in plane cultures or in a roller apparatus within a culture chamber, for hours up to a month. The cultured cells on the titanium surfaces were examined for histology, protein secretion, and gene expression. Results showed that complete osseointegration using human DPSCs was obtained. It was also shown that these cells were capable of differentiating quickly into osteoblasts and endotheliocytes and, then, able to produce bone tissue along the implant surfaces [58]. Osteoblast differentiation of DPSCs and bone morphogenetic protein production was obtained in a better and quicker way, when challenging stem cells with the DMLS titanium surface [58]. These successful results in a short time suggested that DMLS titanium surfaces may represent a promising alternative for clinical use in dental implantology [58].

## 4. Concluding Remarks

In recent years, according to the modern concepts of bone tissue engineering, macroporous structures have been extensively investigated. These porous scaffold materials should be designed to stimulate bone ingrowth so as to enhance implants fixation but must also be able to withstand the load bearing demands.

It is difficult if not impossible to fabricate a titanium scaffold with controlled porosity and open pore internal architecture via conventional manufacturing routes. AM techniques such as DMLS can provide complete control over the microarchitecture of porous titanium implants. This enables the possibility of tailoring and optimizing the structural and mechanical properties of the implants, while maintaining the required pore dimensions that allow for bone and vessel ingrowth.

A number of studies have demonstrated the potential for the use of DMLS titanium implants. The chemical and physical properties of dental implants fabricated with the DMLS technique have been characterised. The biologic response to the DMLS implant surface has been investigated in different* in vitro* studies, in which human fibrin clot formation and the behaviour of human osteoblasts and mesenchymal stem cells were analyzed. The behaviour of DMLS implants has been investigated* in vivo* in histologic and histomorphometric studies in both animals and humans, and satisfactory outcomes were reported. The first clinical studies on DMLS titanium dental implants have reported satisfactory short-term results.

In all these studies, DMLS implants were designed with a porous surface and a dense titanium core. However, further studies that clearly demonstrate benefits of DMLS implants over conventional implants are needed. In particular, as dental implants are expected to survive long periods, further prospective studies are needed, to investigate the clinical performance of DMLS implants in the long-term; in addition, it would be important to understand better the fatigue mechanical behaviour of implant systems fabricated by DMLS.

Further development and advances in DMLS will require optimal scaffold design and the input of enhanced knowledge of cell physiology, including optimal cell seeding and vascularization; in addition, the application of surface treatments may potentiate the biological response to DMLS titanium implants. Nevertheless, the introduction of DMLS technology signals the start of a new revolutionary era for implant dentistry as its immense potential for producing highly complex macro- and microstructures is receiving considerable interest in a wide variety of medical fields.

## Appendix

### Modified NOS Scale Adapted for This Review

*Note*. A study can be awarded a maximum of one star (^∗^) for each numbered item within each category.

*Selection*

(1) Sample size calculation
  (a) yes^∗^
  (b) no
(2) Representativeness of the patients treated with* direct metal laser sintering titanium dental implants* (either “a” or “b” here can give a star)
  (a) truly representative of the average sample of patients treated in the clinical centre^∗^
  (b) somewhat representative of the average sample of patients treated in the clinical centre^∗^
  (c) selected group of patients
  (d) no description of the derivation of the group
(3) Selection of the patients treated with other types of implants
  (a) drawn from the same community as the patients submitted to immediate/early loading protocols^∗^
  (b) drawn from a different source
  (c) no description of the derivation of the patients
  (d) not included in the study
(4) Ascertainment/assessment of peri-implant conditions
  (a) adequate (based on probing measurements, i.e., PPD and CAL, radiographic evaluation, or histological analysis)^∗^
  (b) inadequate (use of nonprobing evaluations, i.e., self-reported implant loss and peri-implant diagnosis based on other methods)
  (c) unclear (methods were not clear or not reported)
(5) Clear definitions of study protocol applied
  (a) yes^∗^
  (b) no
(6) Training/calibration of assessors of clinical outcomes
  (a) yes^∗^
  (b) no/not reported
(7) Prospective data collection and description of clear inclusion/exclusion criteria
  (a) yes^∗^
  (b) no

*Comparability*

(1) Comparability of groups (patients) on the basis of the design or analysis
  (a) all patients received similar implant therapy^∗^
  (b) not all patients received similar implant therapy
(2) Management of confounders (data collection and investigation of impact)
  (a) study/assessment performed with control for confounders^∗^
  (b) study/assessment performed without control for cofounders (unadjusted analysis)

*Outcome*

(1) Assessment of peri-implant outcomes
  (a) independent blind assessment^∗^
  (b) nonblinded assessment
  (c) self-report
  (d) no description
(2) Ascertainment/criteria applied to assess the outcomes of* direct laser metal sintering titanium dental implants*
  (a) adequate (performed in the clinical centre and based on clinical/radiographic/histological outcomes)^∗^
  (b) inadequate (performed outside the clinical centre/self-reported)
  (c) unclear (methods were not clear or not reported)
(3) Adequacy of follow-up of patients
  (a) complete follow-up, all subjects accounted for^∗^
  (b) subjects lost to follow-up unlikely to introduce bias, small number lost and ≥70% follow-up, or description provided of those lost)^∗^
  (c) follow-up rate <70% and/or no description of those lost
  (d) no statement

*Statistics*

(1) Appropriateness/validity of statistical analysis
  (a) valid^∗^
  (b) invalid
  (c) unclear or not reported
(2) Unit of analysis (response rate)
  (a) number of patients and/or implants per group^∗^
  (b) percentage of patients and/or implants per group
  (c) unclear or not reported.
